# Trochleoplasty procedures show complication rates similar to other patellar-stabilizing procedures

**DOI:** 10.1007/s00167-017-4766-5

**Published:** 2017-12-05

**Authors:** Jordy D. P. van Sambeeck, Sebastiaan A. W. van de Groes, Nico Verdonschot, Gerjon Hannink

**Affiliations:** Department of Orthopaedics, Radboud University Medical Center, PO Box 9101, 6500 HB Nijmegen, The Netherlands

**Keywords:** Patellofemoral instability, Trochlear dysplasia, Trochleoplasty, Trochlear osteotomy, Complications

## Abstract

**Purpose:**

Trochleoplasty aims to restore patellar stability. Various techniques have been described and almost all authors report successful results. However, the procedure has a significant risk of complications. Purpose of this study was to perform a systematic review and meta-analysis of the available literature to assess the rate of complications after the various techniques used for trochleoplasty procedures.

**Materials and methods:**

MEDLINE, EMBASE, Web of Science and Cochrane Library databases were searched. Studies on patients with recurrent patellar instability treated with a trochleoplasty with or without additional procedure, and reported complications were included. The primary outcome was the rate of complications per technique. A meta-analysis was performed whenever three or more studies per surgical technique could be included.

**Results:**

The selection process resulted in 20 studies included for analysis. A lateral facet elevating trochlear osteotomy was reported by two studies, ten studies reported on a Bereiter trochleoplasty, five on a Dejour trochleoplasty, one on an arthroscopic technique, one on a ‘modified’ technique and one on a recession wedge trochleoplasty. Meta-analysis showed that proportion of recurrent dislocation was 0.04 (95% CI 0.02–0.07) for Bereiter trochleoplasty and 0.02 (95% CI 0–0.08) for Dejour trochleoplasty. These proportions were 0.06 (95% CI 0.02–0.13) and 0.09 (95% CI 0.03–0.27) for recurrent instability, 0.07 (95% CI 0.02–0.19) and 0.12 (95% CI 0.00–0.91) for patellofemoral osteoarthritis and 0.08 (95% CI 0.04–0.14) and 0.20 (95% CI 0.11–0.32) for further surgery respectively.

**Conclusion:**

This study demonstrates that the complications after a Bereiter and Dejour trochleoplasty including additional procedures are in the range of those of other patellar stabilizing procedures. For four other techniques, no meta-analysis could be performed. The clinical relevance of this study is that it provides clinicians with the best currently available evidence on the rate of complications after trochleoplasty procedures. This can be helpful in the process of deciding whether or not to perform such a procedure, and can be used to better inform patients about the advantages and disadvantages of different trochleoplasty procedures.

**Level of evidence:**

Level IV.

## Introduction

Patellar dislocation occurs when the patella completely disengages from the trochlear groove. The most common recurrent symptom after patellar dislocation is patellar instability, which includes both patellar dislocation and subluxation [31]. Trochlear dysplasia has been identified as the most consistent anatomic factor present in patients with recurrent patellar dislocations [24].

Trochleoplasty is a surgical procedure designed to reshape the trochlea in patients with recurrent patellar dislocation and trochlear dysplasia. Trochleoplasty involves working directly on the patellofemoral joint, modifying the congruency between the two articulating bones and alteration of joint kinematics, with a high risk of cartilage damage. The number of trochleoplasty procedures as a primary or revision surgical treatment option in patients with recurrent patellar dislocation and trochlear dysplasia has increased over the last decade [25]. Most authors agree that trochleoplasty procedures should always be combined with soft-tissue and/or with bony procedures (e.g. medial patellofemoral ligament (MPFL) reconstruction (lowest rate of recurrence with double-limb graft configuration [37]), tibial tubercle transposition) as indicated. Therefore, a trochleoplasty procedure could be defined as a trochleoplasty including any additional stabilizing procedure.

Various techniques for trochleoplasty have been described in the past decades. Four basic trochleoplasty procedures can be distinguished: (1) the lateral-facet elevating trochleoplasty as first described by Albee [1], (2) the sulcus-deepening trochleoplasty which was first proposed by Masse [18] and later modified by Dejour [11], (3) the ‘Bereiter’ or ‘thin-flap’ procedure [6], and (4) the ‘recession’ or ‘recession-wedge’ trochleoplasty [15]. Although, the outcome measures vary widely between individual studies (e.g. Kujala Knee Score, Lysholm Score, Knee Injury and Osteoarthritis Outcome Score, etc.), most articles present satisfactory results of trochleoplasty procedures in creating a stable patellofemoral joint in terms of recurrence of patellar dislocation. However, complications are often not included as primary outcome measure but are only briefly described within the “Results” or “Discussion” in general terms. Patellar redislocation as a complication is rarely reported [25], however postoperative stiffness and return to the operating room for any reason are relatively frequent reported complications.

Trochleoplasty is a highly complex surgical technique with a significant risk for complications [17, 25, 36]. Therefore, it is important to gain more knowledge on complications after trochleoplasty procedures. To assess the rate of complications after the various techniques used for trochleoplasty procedures a systematic review and meta-analysis of the available literature was performed. The results of this study provide clinicians with the best currently available evidence on the rate of complications after a trochleoplasty procedure. This can be helpful to properly inform the patient and to make a well-informed decision as to whether or not to perform this procedure.

## Materials and methods

A systematic review was conducted and reported in accordance with the reporting guidance provided in the Preferred Reporting Items for Systematic Reviews and Meta-Analyses (PRISMA) statement [21]. The protocol was prospectively registered in PROSPERO (https://www.crd.york.ac.uk/PROSPERO/display_record.asp?ID=CRD42015029815).

### Search

MEDLINE, EMBASE, Web of Science and Cochrane Library databases were searched (last search performed 10 May 2016). The search strategy was determined in collaboration with an information specialist from the medical library of the Radboud University Medical Center. Keywords used to develop our search strategy were ‘patellar instability’, ‘trochleoplasty’, and ‘complications’. The detailed search strategy is provided in "Appendix”. Reference lists of included studies and relevant reviews were screened for relevant studies. No Grey literature search was undertaken.

### Eligibility and Study selection

All articles were screened based on title and abstract by two reviewers (JvS, SvdG). In this screening stage, studies were excluded if they fulfilled 1 of the following criteria: (1) no trochleoplasty performed; (2) no clinical outcome study on humans (observational and/or experimental) or description of operative technique; (3) animal study, case report, review article, cadaveric study, in vitro study, biomechanical study or conference proceeding; (4) article not in English, Dutch, French, or German (all languages were screened); (5) article published before 1990. In the subsequent full text screening stage studies were further evaluated for eligibility. Studies were excluded if they met any of criteria 1–5 or 1 of the following: (6) no report of complications; (7) indication for trochleoplasty was not recurrent patellar instability. In addition, studies were excluded if they contained data also published in another included paper. In case of a study being part of a larger, original study, the original study was included. In case of reported preliminary data the most extended paper was included in the analysis. Discrepancies between the reviewers were resolved by discussion and consensus.

The primary outcome was the rate of complications of trochleoplasty procedures. Complications were defined as: a negative outcome including returning to the operating room (OR), symptomatic hardware, loss of range of motion (ROM), increased pain/apprehension leading to return to the OR, patella redislocation/subluxation/instability, accelerated (radiological) progression of patellofemoral osteoarthritis (PF OA), deep venous thrombosis (DVT), infection, distal femoral fracture. Complications were subdivided in minor or major complications. Minor complications included complaints of recurrence of maltracking or subluxation, loss of up to 20° ROM not requiring surgical treatment, increase in PF OA to grade 2 or 3 according to Iwano classification, superficial wound infection, anesthetic complications. Major complications included redislocation of the patella, return to OR due to increase in pain or recurrence of instability or any other cause, reduced ROM requiring arthrolysis, hardware removal because of pain or crepitus, progression to grade 4 PF OA, venous thrombotic event. Residual pain, swelling or crepitus not leading to OR were considered outcomes of the procedure and not complications.

### Data collection and analysis

Data were extracted from the included articles by two reviewers (JvS, SvdG) and included: study ID, number of patients, number of knees, type of trochlear dysplasia, duration of symptoms, indication for surgery, mean patient age at surgery, patient sex, previous surgery on the involved knee, type of trochleoplasty performed, additional procedures performed, type and rate of complications and (if mentioned) time when complication occurred, length of follow-up and patients lost to follow-up. In studies that reported only percentages of complications and no absolute numbers, absolute numbers of complications were calculated based on the number of patients or surgical procedures reported. Subsequently, a meta-analysis was performed whenever three or more studies per surgical technique that reported on a type of complication could be included. Despite anticipated heterogeneity, the individual study proportions were pooled. Pooled estimates of proportions with their corresponding 95% confidence intervals were calculated using Freeman–Tukey double arcsine transformation within a random effects model framework. Heterogeneity of combined study results was assessed by *I* ^2^, and its connected Chi-square test for heterogeneity, and the corresponding 95% confidence intervals were calculated. Restricted maximum likelihood was used to estimate the heterogeneity variance. Statistical analyses were performed using R version 3.4.0 (R Foundation for Statistical Computing, Vienna, Austria) with package ‘meta’.

### Quality assessment

Quality assessment was not performed as the included articles were retrospective or prospective single-arm cohort studies and no validated scores for the methodological quality of these type of studies are available.

## Results

**Fig. 1:**
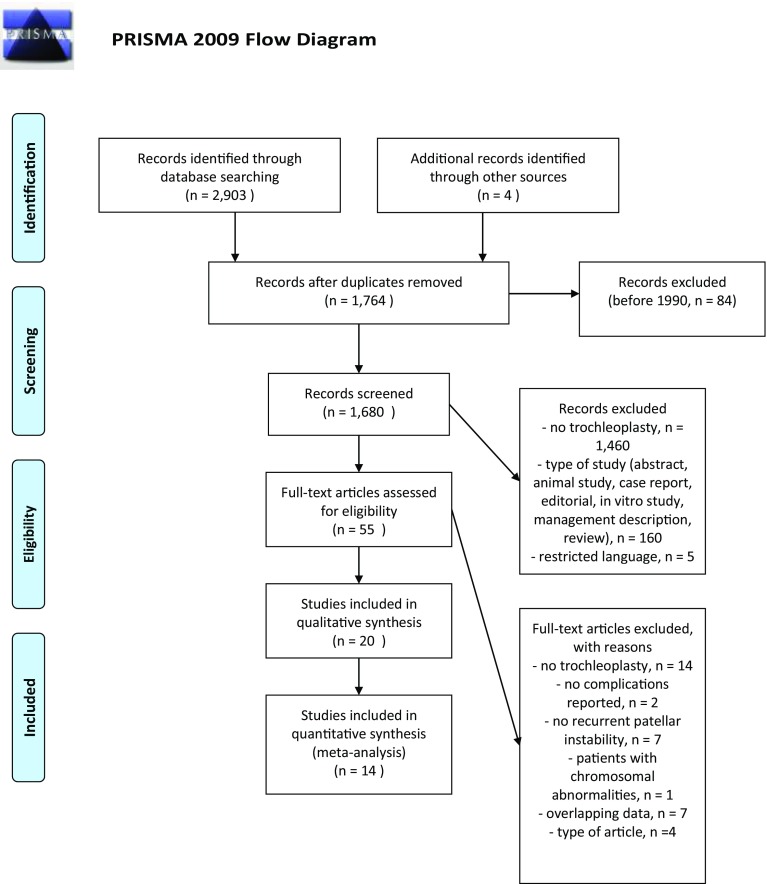
PRISMA flow diagram

The search strategy retrieved 1,848 unique records. Subsequent selection procedure resulted in 55 eligible articles of which 20 studies could be included in this systematic review (Fig. 1).

Table 1 displays study characteristics including population description, type of trochleoplasty performed, additional procedures performed, and the number of complications.

**Table 1:** Study characteristics

Author Year	Nr. of patients included	Nr. of knees	Duration symptoms before surgery	Mean age surgery in years (range)	Sex (% female)	Follow up (mean in months)	Nr. knees lost to FU	Type trochleo-plasty	Tibial tubercle transfer	Medial soft tissue procedure	Miscellaneous	Post-operative complication related to trochlea-plasty (nr. of patients)	Major complications (number; percentage)	Minor complications (number; percentage)
Lateral facet elevating trochlear osteotomy														
Badhe et al. (2003)	4	4	Long history	32 (24–38)	75	12	0	Albee	0	0	4 patellar osteotomy	10–20 degrees loss of flexion (4)	0; 0%	4; 100%
Koëter et al. (2007)	16	19	N/A	25 (15–34)	N/A	51	0	Modified Albee	0	0	–	Postoperatieve haematoma that had to be evacuated (1) 1 grade progression of osteoarthritis (2) Subluxation after rotation trauma (2), 1 undergoing reposition of the tibial tubercle which was transposed in a former procedure Persisting pain treated with patellofemoral arthroplasty (1)	3; 16%	3; 16%
Bereiter trochleoplasty														
Banke et al. (2014)	17	18	N/A	22,2	65	30,5	0	Bereiter	0	18 MPFL	–	Medial subluxation undergoing patella readjustment (1) Reduced ROM undergoing early arthroscopic arthrolysis (2)	3; 17%	0; 0%
Camathias et al. (2016)	44	50	> 6 months	15,6 (13–20)	60	33	0	Modified Bereiter	0	0	–	Spontaneous redislocation (1) Arthrofibrosis requiring arthroscopic arthrolysis (4)	5; 10%	0; 0%
Fucentese et al. (2011)	38	44	N/A	18 (median) (14–40)	75	48 (median)	4	Deepening Bereiter	0	44 VMO	–	Transient postoperative femoral nerve palsy after peripheral anesthesia (1) Wound healing problem (1) Complex regional pain syndrome (1) Ongoing pain undergoing arthroscopic debridement with removal of loose bodies (3) Ongoing sensation of instability during activities of daily living (6), 1 undergoing MPFL reconstruction and 1 anteromedialization of the tibial tubercle Recurrent atraumatic dislocation (1)	6; 14%	7; 16%
Metcalfe (2015)	185	195	N/A	21	72	Minimum 1 year	19	Deepening Bereiter	N/A	N/A	–	Ongoing dislocations (16)	16; 8%	0; 0%
Nelitz et al. (2013)	23	26	N/A	19,2 (15–23)	38	30	N/A	Deepening Bereiter	0	26	–	No complications reported	0; 0%	0; 0%
Schöttle et al. (2005)	16	19	N/A	22 (17–40)	81	36	0	Deepening Bereiter	0	19 VMO	–	Increased degenerative changes of the trochlea (1)	0; 0%	1; 5%
Utting et al. (2008)	54	59	Mean 7 years	21,5 (14.3–33.9)	72	24	13	Deepening Bereiter	4	14 MPFL and VMO 5 VMO 4 MPFL	–	Superficial wound infection (2) Manipulation under anesthesia (1) Traumatic dislocation (1) Anaphylactic reaction after administration of prophylactic antibiotic on induction of anesthesia (1) Recurrence of symptoms (10)	2; 3%	13; 22%
Von Knoch et al. (2006)	38	45	N/A	22,2 (15–31)	58	99,6	3	Deepening Bereiter	0	45, MPFL as required	–	Patella baja (1) Subluxations undergoing additional Elmslie-Trillat procedure (1) Progression of PF OA to Iwano grade 1 (14), 2 (7), 3 (2) or 4 (1)	2; 4%	24; 96%
Neuman et al. (2014)	42	46	N/A	27,6 (median) (16–53)	72	56,7 (median)	20	Deepening Modified Bereiter	0	46 VMO and MPFL	–	Radiological progression of PF OA (3)	0; 0%	3; 7%
Bereiter and Gautier (1994)	10	12	N/A	20 (15–30)	70	24	6	Bereiter	N/A	N/A	–	Postoperative bleeding (1) Algodystrophy (1)	0; 0%	2; 17%
Arthroscopic deepening trochleoplasty														
Blond and Haugegaard (2014)	31	37	9–348 months	19 (median) (12–39)	68	12–57 (range)	0	Arthroscopic deepening	0	37 MPFL	–	Pronounced anterior knee pain at flexion undergoing lateral release (3) Symptomatic subluxations corrected by medialization of the tibial tubercle (2)	5; 14%	0; 0%
Dejour trochleoplasty														
Dejour and Ntagiopoulos (2013)	22	24	N/A	23 (14–33)	75	66,5	0	Deepening (Dejour)	12	11 MPFL 10 VMO	6 lateral release 1 patellar osteotomy 4 PT lengthening & proximal TT transfer	No complications reported	0; 0%	0; 0%
Faruqui et al. (2012)	6	6	N/A	21,5 (15–38)	83	68,3	6	Deepening	3	3 MPFL 2 imbrication	–	Mentioning of complications absent	0; 0%	0; 0%
McNamara et al. (2015)	90	107	N/A	23 (12–49)	60	72	N/A	Deepening (modified Dejour)	11	14 MPFL	16 patello-plasty 28 lateral release	Venous thrombotic event (2: 1 DVT, 1 PE) Superficial wound infection (4) Complaints of significant crepitus (4), 2 underwent patelloplasty Continuing instability symptoms undergoing MPFL-reconstruction (10) Arthroscopic arthrolysis (7), open arthrolysis (1) Removal of loose absorbable screw heads (2) Arthroscopic debridemnt of a notch “osteophyte” (1)	24; 23%	7; 6,5%
Ntagiopoulos et al. (2013)	27	31	N/A	21 (14–47)	48	94	0	Deepening (Dejour)	21–31	5 MPFL 26 VMO	21 lateral release	Hardware breakage that had to be removed by arthroscopic surgery (2) Deep venous thrombosis (1)	2; 7%	1; 3,2%
Rouanet et al. (2015)	34	34	N/A	27.8 (16–49)	71	183.6	11	Sulcus deepening	17	34 Insall procedure		Postoperative stiffness at < 90° flexion requiring manipulation under anesthesia (6) or arthroscopic release (2) Pain and Iwano stage 4 PF OA undergoing total knee arthroplasty (3) or patellofemoral arthroplasty (3) Pain and frequently giving out of the knee requiring anterior tibial tubercle transfer (1) Occasional instability (10) Progression of PF OA to Iwano stage ≥ 2 (22)	15; 44%	32; 94%
Modified deepening trochleoplasty														
Zaki and Rae (2010)	25	27	N/A	25 (19–36)	72	54	N/A	Deepening	5	3	14 medial reefing + Roux procedure (8 + VMO)	Superficial wound infection (3)	0; 0%	3; 11%
Recession wedge trochleoplasty														
Thaunat et al. (2011)	17	19	Mean 11 years	23 (18–45)	56	34	1	Recession wedge	18	8 MPFL	19 lateral release	Knee stiffness requiring arthroscopic arthrolysis (1) Painful persistent ridge requiring arthroscopic supratrochlear exostosectomy (1) Traumatic dislocation (1) Recurrence of instability (1) Progression of PF OA to Iwano stage 2 (3)	3; 16%	4; 21%

Trochleoplasty procedures were performed on 822 knees in 739 patients. Average age of the patients was 22.6 years (range 12–53 years). Sixty-seven percent of patients were female. Mean follow-up was 57 months, mean follow-up in individual studies ranged from 12 months to 183 months (16 studies reported mean, 2 medians, 1 range and 1 a minimum of 1 year).

Indications for trochleoplasty were recurrent patellar instability, defined as at least two patellar dislocations (in 1 study based on one documented patellar dislocation [19]), with underlying trochlear dysplasia. Ten studies [5, 7, 9, 14, 19, 24, 28, 29, 33, 38] reported trochlear dysplasia defined according to the Dejour classification of trochlear dysplasia [10] on conventional X-rays or MRI. In two studies, an elevated trochlear boss height on X-ray was additionally required as indication [19, 38]. For some studies, indication was also based on presence of the apprehension sign or lateral patellar glide test [5, 7, 14, 24, 29, 35].

All studies reported that additional procedures were performed, except the one of Bereiter [6] that did not report on additional procedures. On average, 46% of patients had undergone previous procedures before trochleoplasty including modified Fulkerson–Elmsie Trillat osteotomy, diagnostic arthroscopy, arthroscopic/open lateral release, tibial tubercle transfer, VMO-plasty, Roux–Goldthwaite procedure and chondroplasty.

A total of 190 complications occurred in 822 knees, including recurrence of instability (subluxation and dislocation), loss of knee range of motion, development or progression of PF OA, return to OR and miscellaneous surgical complications, such as wound complications.

A lateral facet elevating trochlear osteotomy was reported by two studies [3, 16]. A deepening trochleoplasty was reported by 17 studies: ten reported on a (modified) Bereiter trochleoplasty [5, 6, 8, 14, 20, 22, 23, 29, 34, 35], five on a (modified) Dejour trochleoplasty [9, 12, 19, 24, 28], one on an arthroscopic technique [7], and one on a ‘modified’ technique [38]. A recession wedge trochleoplasty was reported by one study [33].

### Complications and miscellaneous results of techniques included in the meta-analysis

**Fig. 2:**
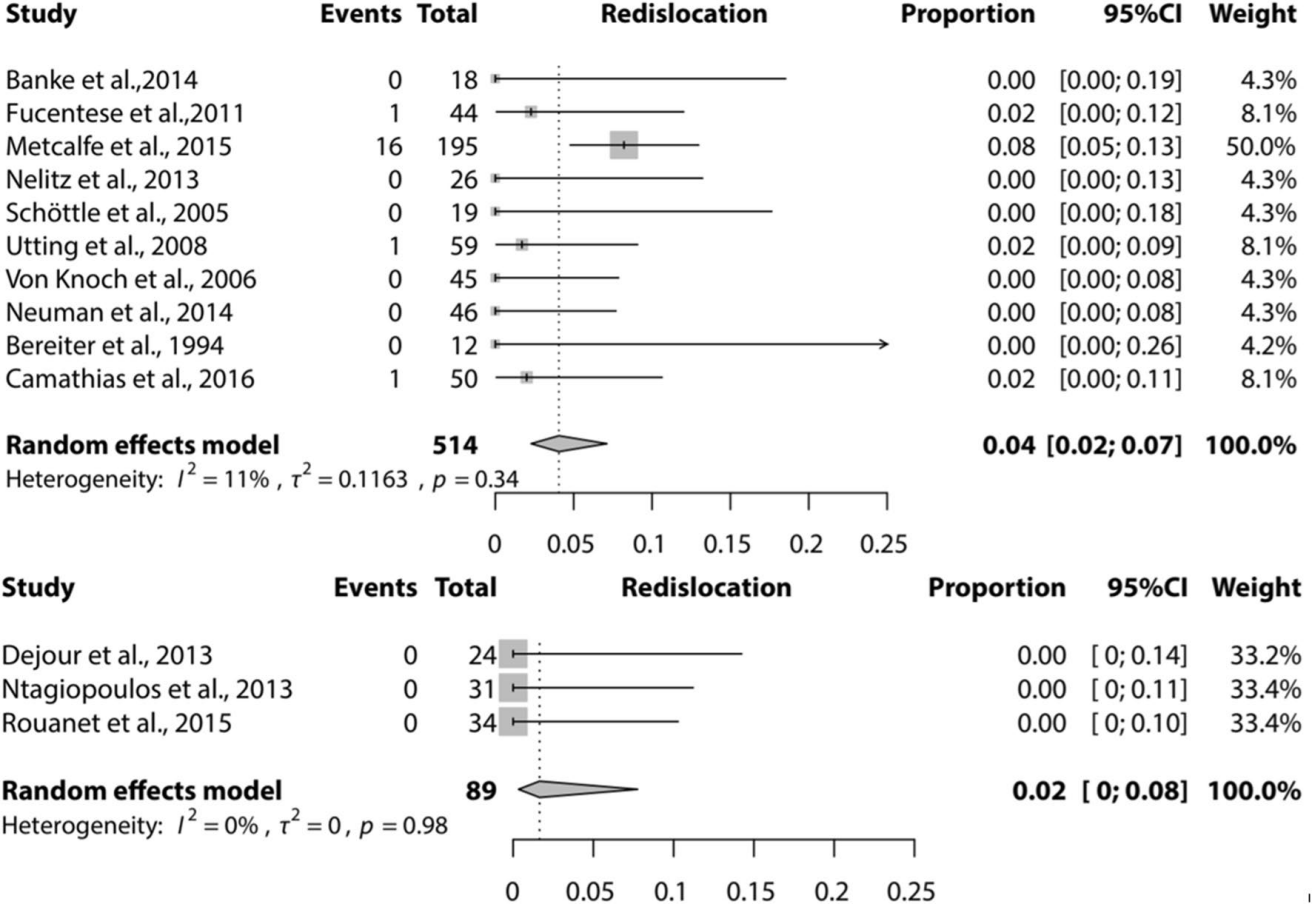
Forest plot of proportion of recurrent dislocation after a Bereiter trochleoplasty (upper) and Dejour trochleoplasty (lower)

**Fig. 3:**
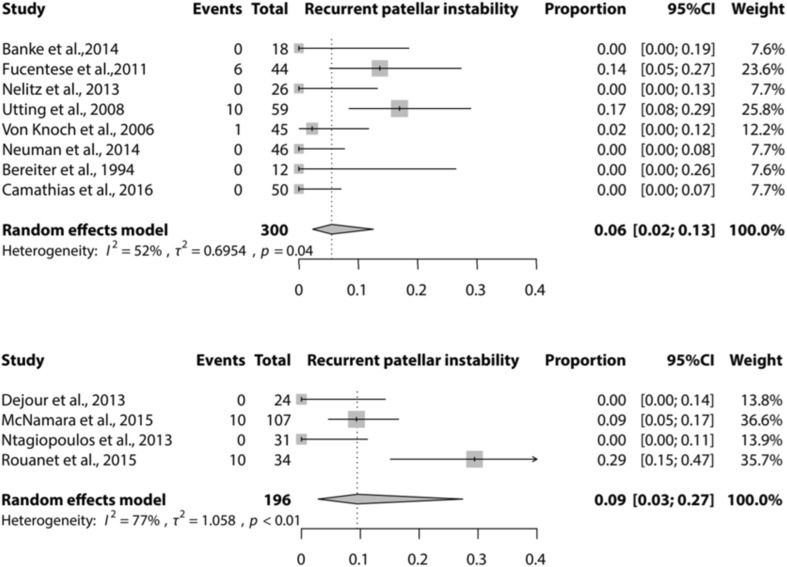
Forest plot of proportion of recurrent patellar instability after a Bereiter trochleoplasty (upper) and Dejour trochleoplasty (lower)

**Fig. 4:**
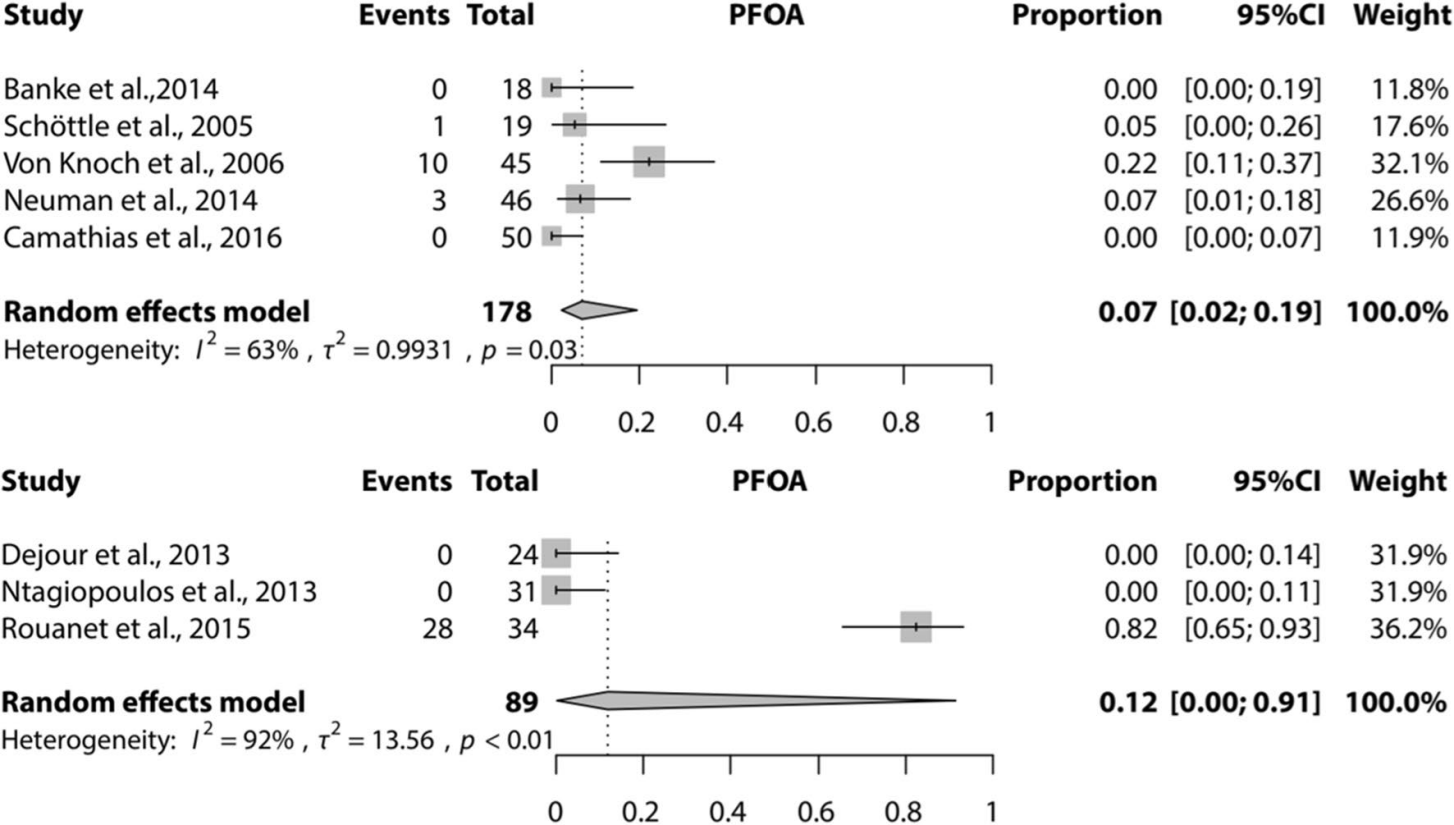
Forest plot of proportion of patellofemoral osteoarthritis (PF OA) after a Bereiter trochleoplasty (upper) and Dejour trochleoplasty (lower)

**Fig. 5:**
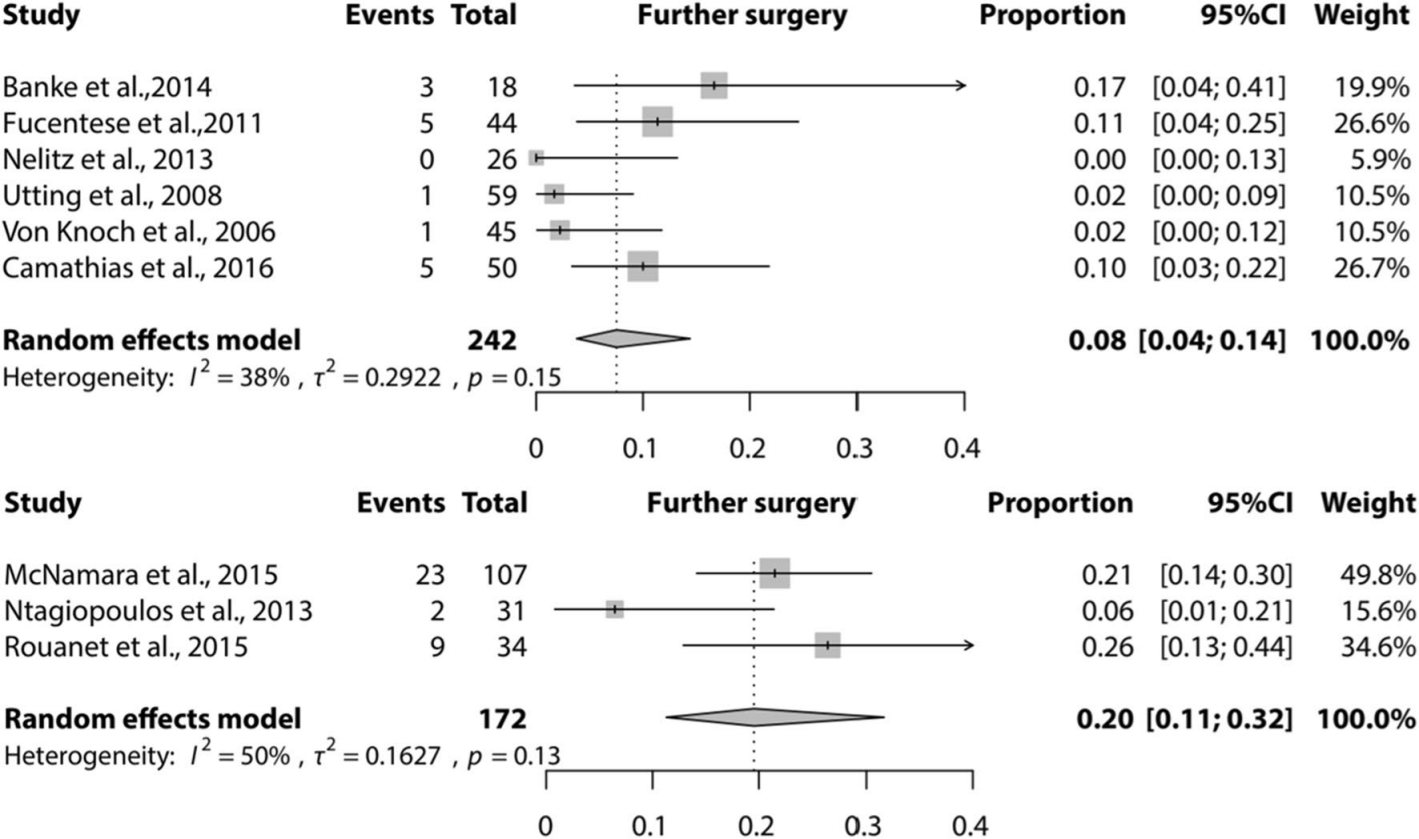
Forest plot of proportion of patients who needed further surgery after a Bereiter trochleoplasty (upper) and Dejour trochleoplasty (lower)

**Fig. 6:**
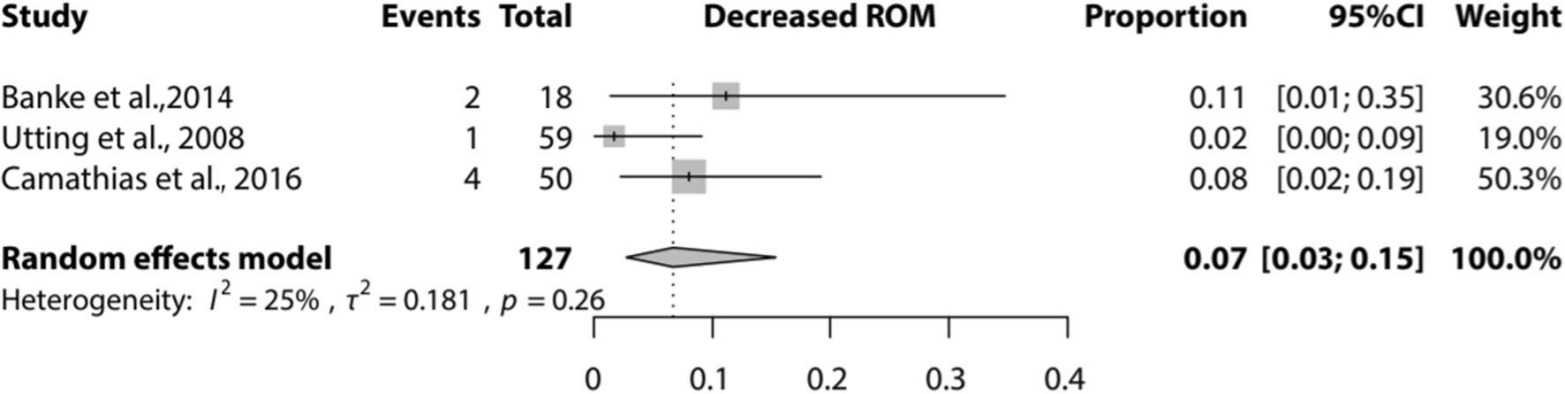
Forest plot of proportion of patients with loss of range of motion after a Bereiter trochleoplasty

Meta-analysis could be performed for the complications recurrence of patellar instability (subluxations), recurrent dislocation, PF OA, and further surgery needed for the Dejour and Bereiter trochleoplasty techniques only. Meta-analysis for loss of ROM could only be performed for the Bereiter trochleoplasty. Figures 2, 3, 4 and 5 show the results of the meta-analyses, including proportion of patients with recurrent dislocation (Fig. 2), recurrent instability (Fig. 3), PF OA (Fig. 4) and need for further surgery (Fig. 5). The indications for further surgery were not included in the meta-analysis. For the Bereiter trochleoplasty these were medial subluxation in one patient, reduced ROM in six patients, persistent pain in three patients and recurrence of instability in three patients. For the Dejour trochleoplasty these numbers were complaints of crepitus in two patients, recurrence of instability in ten patients, reduced ROM in 24 patients, persistent pain in one patient, PF OA in six patients, loose absorbable screw heads in two patients, hardware breakage in two patients and a trochlear notch osteophyte in one patient. The proportion of patients with loss of ROM which needed intervention is shown in Fig. 6.

Included in the major complications but not included in the meta-analysis is a pulmonary embolus in one patient in the study of McNamara et al. [19] Minor complications not included in the meta-analysis are a superficial wound infection in two patients in the study of Utting [34] and in four patients in the study of McNamara [19], a deep venous thrombosis in two patients [19, 24], a complication related to anesthesia in two patients [14, 34], a wound healing problem in one patient [14], a complex regional pain syndrome in two patients [6, 14] and a postoperative bleeding in one patient [6]. Fifty-eight patients had unchanged or increased pain not requiring reoperation, 95 patients have some residual symptoms such as clicking, swelling or pain. Six patients kept complaints of crepitus without further surgical treatment and 14 patients kept complaints of swelling.

### Complications and miscellaneous results of techniques not included in the meta-analysis

Two studies reported on a lateral facet elevating trochlear osteotomy, one study reported an arthroscopic deepening trochleoplasty, one a modified deepening trochleoplasty, and one a recession wedge trochleoplasty (Table 1).

## Discussion

The most important finding of this study was that Bereiter and Dejour trochleoplasty procedures show complication rates similar to other patellar stabilizing procedures. The rates of reoperation after a Bereiter and Dejour trochleoplasty [0.08 (95% CI 0.04; 0.14) and 0.20 (95% CI 0.11; 0.32)] are comparable with those found in other systematic reviews of patellar stabilizing procedures (4.1% after MPFL-reconstruction [30], 18% after tibial tubercle osteotomy [27] and 25% after trochleoplasty versus 7% after MPFL-reconstruction [32]). Decreased range of motion and recurrence of instability were the two most frequent reasons for further surgery. The study of McNamara et al. [19] largely contributed (23 patients) to the number of patients returning to the OR after a Dejour trochleoplasty. Ten of these patients underwent an additional MPFL reconstruction and eight underwent arthrolysis. Seven of the eight patients undergoing arthrolysis were from their early cohort of patients before the continuous passive motion was introduced. This study of McNamara et al. might, therefore, confound the rate of reoperation after a Dejour trochleoplasty.

The proportion of recurrent dislocation after a Bereiter or Dejour trochleoplasty [0.04 (95% CI 0.02–0.07) and 0.02 (95% CI 0–0.08)] was lower than or equal with previous results in literature [4, 26, 30]. In their systematic review, Smith et al. [31] found 13% recurrent patellar dislocations after 2–5 years follow-up after surgical intervention for patellar dislocation. Meta-analysis showed that the proportion of recurrence of instability (sensation of instability or subluxation) was low for Bereiter [0.06 (95% CI 0.02–0.13)] and Dejour [0.09 (95% CI 0.03–0.27)] trochleoplasty. This is low compared with the natural course after patellar dislocation, or patients treated non-surgically being up to 24% according to Smith et al. [2, 13, 31]. From these results, it could be hypothesized that these two trochleoplasty techniques are successful in preventing recurrent dislocation and/or instability symptoms, also compared with other surgical interventions.

Seven studies did not report about the presence of PF OA. The rate of development of PF OA would probably increase at longer follow-up, as the development and progression of PF OA in these patients depends on multiple factors, not only a stable patella. Registration of patellofemoral osteoarthritic changes on imaging does not mean that patients have complaints related to PF OA. The number of PF OA should be interpreted as an objective outcome measure and not as a clinically relevant outcome measure if it is asymptomatic. Most of the studies included in this review were not designed to detect PF OA as an outcome measure. The proportion presented in our results could be an underestimation of the true incidence of PF OA and should be interpreted with caution.

Rare complications that were reported include medial subluxation [5], patella baja [35] and venous thrombotic events [19, 24], none were catastrophic. There was no mortality associated with trochleoplasty. One should be aware that these and potential other rare complications can occur after a trochleoplasty since it is a very complex procedure.

Some potential limitations of this study have to be discussed. Since no comparative studies are included, no direct comparison between different techniques could be made. No conclusion can be drawn as to whether one of the techniques is superior to the other in terms of complications of surgery. Furthermore, there is no clear consensus on the indication for trochleoplasty surgery, which makes a direct comparison between studies and/or techniques very difficult. The presented complications for different techniques should be interpreted in the context of the individual studies that have been published, including exact indication for surgery, duration and severity of symptoms, and patient factors.

The definition of complications is always arguable and will differ between different clinicians and patients. Mild residual symptoms such as pain, swelling or clicking were classified as an outcome of surgery and not as a complication of surgery. Some complications cannot be definitely assigned to either the trochleoplasty or the additional procedure, this introduces most likely some bias in complication rate.

It should be noted that the absence of complications does not mean that a patient is free of complaints. The rate of complications found in this review is acceptable, but trochleoplasty is still a rather radical surgical procedure with significant risks.

Almost all studies were retrospective or prospective case series. None of the studies were randomized or described a difference between two cohorts. Because of this lack of methodological quality, we did not perform a quality assessment; all studies were regarded low-level evidence.

Publication bias may be present since “negative” results of case series of surgical procedures are less likely to be submitted for publication. Measurement bias may have occurred due to the failure of thorough administration of complications, especially for minor complications in retrospective studies also due to diligence and increased awareness of the screening resulting in higher report of complications.

There might also be sampling bias, since most surgeons who performed trochleoplasty in the articles in this review were experienced surgeons, thus the number of complications might be an underestimation of the true number.

With the limited high-quality evidence available, we think the results of this study a sufficiently accurate represent the complication rate after trochleoplasty procedures including any additional procedures.

## Conclusions

This systematic review and meta-analysis demonstrates that the complications after a Bereiter and Dejour trochleoplasty including additional procedures are in the range of those of other patellar stabilizing procedures. For four other techniques, no meta-analysis could be performed.

## Appendix

### Pubmed search

((“Patellar Dislocation”[Mesh] OR (“Patella”[Mesh] AND “Dislocations”[Mesh]) OR ((patella*[tw] OR patello*[tw] OR trochlea*[tw]) AND (dislocat*[tw] OR instability[tw] OR instabilities[tw] OR instable[tw] OR luxation*[tw] OR subluxation*[tw]))) AND (trochleo*[tw] OR trochlea*[tw] OR Sulcus[tw] OR patellar groove[tw] OR patellar dislocation/surgery)) OR patellar dislocation/complications.

### Embase and Web of Science search terms

((Patella dislocation/OR (exp patella/AND exp dislocation)) OR ((patella* or patella* or trochlea) AND (dislocate* or instability or instabilities or instable or luxation* or subluxation*))ti.ab.kw.) AND ((trochlea* or trochleo*).ti.ab.kw. OR sulcus ti.ab.kw. OR patella dislocation/surgery) OR patella dislocation/complication. Limits: conference abstract or conference proceeding.

### Cochrane Library search

Patellar Dislocation [MeSH] OR (Patella [MeSH] AND Dislocations [MeSH]) OR ((patella* or patella* or trochlea) AND (dislocate* or instability or instabilities or instable or luxation* or subluxation*)).

## Abbreviations

CPM: Continuous passive motion
DVT: Deep venous thrombosis
MPFL: Medial patellofemoral ligament
MRI: Magnetic resonance imaging
OR: Operating room
PE: Pulmonary embolism
PF OA: Patellofemoral osteoarthritis
PRISMA: Preferred reporting items for systematic reviews and meta-analyses
ROM: Range of motion
VMO: Vastus medialis obliquus
